# Weaning from intravenous prostanoids and normalization of hemodynamics by long-term imatinib therapy in severe idiopathic pulmonary arterial hypertension

**DOI:** 10.1007/s11096-013-9881-x

**Published:** 2013-11-28

**Authors:** Rudolf Speich, Ursula Treder, Guido Domenighetti, Lars C. Huber, Silvia Ulrich

**Affiliations:** University Hospital, Rämistrasse 100, Room HOER C 11, 8091 Zurich, Switzerland

**Keywords:** Imatinib, Pulmonary arterial hypertension, Treatment

## Abstract

*Introduction* Despite new treatment options targeted at its three main pathogenic pathways, prognosis of idiopathic pulmonary arterial hypertension has remained dismal, with 3-year survival rates around 70 %. Antiproliferative agents have emerged as a new therapeutic concept. However, they may exert their effects only after a prolonged period of time. *Case description* Herein we present a patient who, despite being on a triple targeted drug therapy including high-dose intravenous prostanoids, still had severe pulmonary hypertension. After 4 years treatment with the tyrosine kinase inhibitor imatinib, the patient could be weaned from intravenous prostanoids and attained a persistent hemodynamic normalization. *Conclusions* Antiproliferative agents might be a promising new class of drugs in pulmonary arterial hypertension. However, the occurrence of unexpected side effects like the increased incidence of subdural hematomas, has led to the recommendation that at present such an off-label use is strongly discouraged, and that further studies elucidating the risk/benefit ratio of tyrosine kinase inhibitors are clearly needed.

## Impacts on practice

- Antiproliferative agents should for the time being not be used off-label in pulmonary arterial hypertension.
- The risk/benefit ratio of tyrosine kinase inhibitors in arterial hypertension remains to be established.

## Introduction

Presently, there are three classes of approved therapies for idiopathic pulmonary arterial hypertension (IPAH), namely endothelin receptor antagonists, phosphodiesterase-5 inhibitors, and prostanoids. Nonetheless, the prognosis of patients with IPAH has remained dismal, with 3-year survival rates around 70 %.

Although experimentally some antiproliferative properties have been attributed to all of these three drug classes, their main treatment effect is vasodilation. However, except in very early disease, vasodilators remain to be a symptomatic treatment. Only recently, the antiproliferative approach has gained clinical interest because of the emergence of the tyrosine kinase inhibitors. By the first experimental proof of concept study in the rat monocrotaline model, Schermuly et al. [1] could show that the antiproliferative agent imatinib, which targets mainly the pathogenically important platelet-derived growth factor [2], was able to reverse pulmonary hypertension. Shortly thereafter, the same group started imatinib in a patient already receiving all three classes of the therapeutic agents mentioned above but refusing lung transplantation [3]. Six months later, there was a dramatic improvement in hemodynamics, 6-min walking distance (6MWD) and functional class.

In the meanwhile, the phase II trial [4] and the subsequent large randomized trial (IMPRES) [5] could demonstrate that imatinib significantly improves hemodynamics within a period of 6 months.

About 2 years after the first case report by Ghofrani et al. [3], we commenced imatinib treatment in selected patients on an off-label use basis. Herein we present a patient suffering from severe IPAH, who could be successfully weaned from intravenous prostanoids within a period of 5 years and attained a persistent hemodynamic normalization.

## Case description

**Fig. 1:**
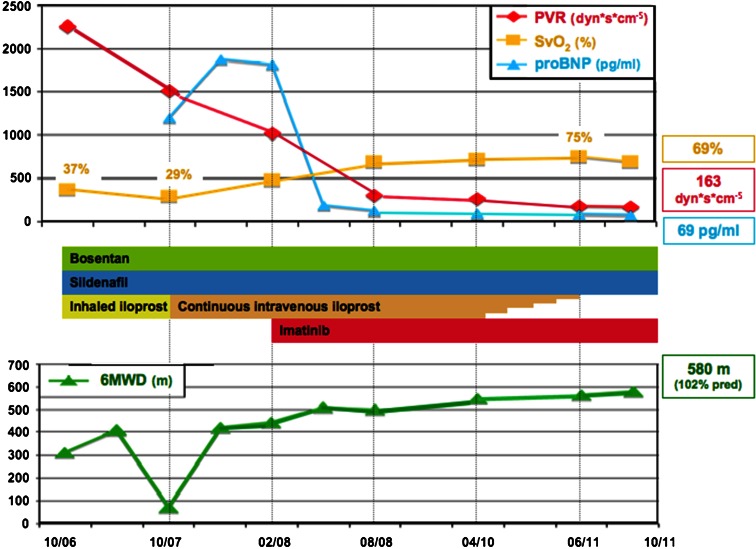
Clinical course of the patient. *PVR* pulmonary vascular resistance, *SvO2* mixed venous saturation, *proBNP* N-terminal pro–brain natriuretic peptide, *6-MWD* 6-minute walking distance

This 37-years-old woman was diagnosed with IPAH in October 2006. She then had a mean pulmonary artery pressure of 68 mmHg, a cardiac output of 1.9 L/min/m^2^, and a pulmonary vascular resistance (PVR) of 2,261 dyn · seconds · cm^−5^ (See Table 1). Chronic thromboembolic, left heart and pulmonary diseases were excluded by negative ventilation/perfusion scan, normal pulmonary artery wedge pressure, and lung function testing. Family history was non-revealing and there were no other diseases known to be associated with PAH. She then was treated with bosentan 125 mg bid., sildenafil 50 mg tid., inhaled iloprost 100 μg daily, oral anticoagulation and diuretic therapy. As depicted in Fig. 1, the 6MWD temporarily improved from 310 to 410 m. However, within 1 year her condition deteriorated dramatically, and in October 2007 the patient became almost bed-ridden. Other causes for this clinical course such as pulmonary embolism could be excluded. Her PVR still was very high (1,506 dyn · seconds · cm^−5^). Treatment with continuous intravenous iloprost was commenced, and its dose was up-titrated to 500 μg per day. This resulted in an improvement in her 6MWD up to 444 m until February 2008. Her hemodynamics, however, were still seriously impaired with a PVR over 1,000 dyn · seconds · cm^−5^. Hence, we started an off-label treatment with imatinib 400 mg per day. Six months later, her pulmonary vascular resistance had decreased to 300 dyn · seconds · cm^−5^.

**Table 1:** Clinical and hemodynamic data over time

	October 2006	October 2007	February 2008	April 2010	October 2011
6-Min walking distance (m)	310	70	444	550	580
WHO functional class	IV	IV	II	II	II
Mean pulmonary artery pressure (mmHg)	68	64	53	27	23
Cardiac index (L/min/m^2^)	1.1	1.7	2.6	4.7	5.7
Pulmonary vascular resistance (dyn · seconds · cm^−5^)	2,261	1,506	1,023	259	163
Pulmonary artery wedge pressure (mmHg)	14	10	5	6	7
Right atrial pressure (mmHg)	16	19	Not done	4	5
Mixed venous oxygen saturation (%)	37	29	48	72	69
N-terminal pro–brain natriuretic peptide (pg/mL)	Not done	1,204	1,822	90	69

During the subsequent 3 years of imatinib therapy, the patient further improved her 6MWD and remained stable in functional class II. Except for an episode of central venous catheter infection there were no complications, adverse events or disease-related events. Since by April 2010 her pulmonary hemodynamics almost normalized, we decided to reduce gradually intravenous iloprost until complete stop by August 2011. Two month later the pulmonary hemodynamics were completely normal.

One year later, the patient still is completely stable on bosentan and sildenafil. Echocardiography showed a normal size of the right atrium and the right ventricle, and a normal right heart function, i.e. a fractional area contraction of 42 % (normal >25) and a tricuspid annular systolic plane excursion of 23 mm (normal >17).

## Discussion

This is to the best of our knowledge the first patient suffering from severe IPAH, who could be weaned from intravenous prostanoids, and completely normalized her pulmonary hemodynamics and right ventricular function, remaining solely on continued dual oral targeted drug treatment. We speculate that this normalization can most probably be attributed to long-term imatinib therapy.

So far, there are only two case series including 17 adults suffering from IPAH, in whom intravenous prostanoids could be discontinued [6, 7]. However, it has to be emphasized that all these patients in fact were switched from prostanoids to the new targeted oral or inhaled therapies becoming available at that time. None of them was already receiving targeted treatments at the start of weaning from intravenous prostanoids. After all, it is noticeable that three patients maintained a normal PVR after transition from intravenous to modern oral or inhaled therapy.

Since the first randomized trial in IPAH 1996, there is an ongoing discussion, what kind of treatment goal is realistic in this otherwise devastating disease. Unfortunately, until now, aiming at a normalization of pulmonary hemodynamics has remained to be a search for the holy grail. None of the two largest series of IPAH patients on long-term epoprostenol has explicitly reported on any patient with normal hemodynamics on last follow-up [8, 9]. The lower 95 % confidence intervals of mean pulmonary artery pressures in these two series were 32 [8] and 40 mmHg [9], respectively. The same was the case even in prognostically favorable patients on long-term calcium-channel blockers with a lower 95 % confidence interval of mean pulmonary artery pressure of 34 mmHg [10].

There are only limited data on the long-term benefits of imatinib [11]. Foreseeably, because of its mode of action a potential breakthrough effect like in our patient might be expected to occur only after some years of tratment. Souza et al. [12] have described two cases, who were on imatinib treatment over a period of 3 years. Both exhibited a sustained hemodynamic improvement. However, at that time their hemodynamics still were far from being normal.

Although not being provable, we strongly believe that the leading effect explaining this extraordinary clinical course of our patient has to be ascribed to a reverse remodeling effect of imatinib. In the rat monocrotaline model, Schermuly et al. [1] found a marked increase in cell proliferation in the pulmonary resistance vessels. This could be explained by a lack of apoptosis in the hypertensive animals comparable to the non-exposed control animals. Treatment with imatinib resulted a significantly increase in apoptosis in the vessel walls, going along with a reduction of the proliferation rate and a near normal vessel morphology. Moreover, these researchers could elegantly show, that platelet-derived growth factor, a known potent inhibitor of apoptosis, which in fact has shown to be increased in the vasculature of patients with IPAH [2], seems to act as the main anti-apoptotic factor in this context. While it was entirely absent in the control animals, it massively increased in the monocrotaline-exposed rats. This increase could almost completely be abrogated by treatment with imatinib.

It could be argued that also the current three targeted therapeutic modalities might have some antiproliferative effect, especially the prostanoids. This has been shown at least in the experimental setting. In humans, however, such a therapeutic effect has never been described so far. Of course, the significance of histological examinations of lungs at autopsy or after explantation is hampered by a negative selection bias including only most severe cases. However, there is one interesting study by Achcar et al. [13], who examined explanted lung from 9 patients who had been treated with prostacyclin for a mean of 40 months, and compared them with lungs from 11 patients who had a similar hemodynamic compromise but other treatments than prostanoids. There was not only no difference in vascular remodeling between the two groups, but even a non-significant trend towards a more pronounced medial thickness and a higher number of plexiform lesions in the prostanoid-treated group. In this context, would like to add another interesting report of a patient, who had been on prostacylin for 18 years, and died because of colonic cancer. At autopsy she still showed an extensive proliferative vasculopathy [14]. Hence, we do not believe that the prostanoid contributed relevantly to the restoration of normal hemodynamics and right ventricular function in our patient.

At this point, a word of caution on the use of imatinib has to be made. The authors of the IMPRES study reported in September 2011 [15], that there were two cases of subdural hematoma while on imatinib during the randomized trial phase, and in addition, another six cases were observed in the extension study, summing up to an incidence of 4.2 % per patient year [11]. When we became aware of this serious complication possibly attributable to imatinib in combination with oral anticoagulation, we immediately took two measures. Firstly, we confronted all current and subsequent patients treated with imatinib with a range of potential morbidity and mortality rates due to subdural hematoma and the potential benefits of imatinib (unpublished data). All patients were willing to take the worst risk–benefit scenario and gave a written informed consent. Secondly, we decided to keep the INR around 2.0. May be due to the latter measure, we did not observe any case of subdural hematoma over a period of currently around 30 patient-years (unpublished data).

Notwithstanding, it has to be stressed that we use imatinib off-label, and this treatment is not in accordance with the current guidelines on management of pulmonary hypertension (4th World Symposium and ERS/ESC guidelines) as well as the recommendations made at the last world symposium on pulmonary hypertension, held in Nice 2013, and the statement made by the authors of the IMPRES trial [5] and an accompanying editorial [16]. The increased incidence of subdural hematomas during imatinib treatment may suggest a poor benefit/risk ratio in PAH. Hence, these authorities stated that until further data are available, the off-label use of imatinib for this indication is strongly discouraged.

Furthermore, tyrosine kinase inhibitors should generally be used with caution in PAH since they may have other unexpected side effects. This is exemplified by reports on the development of PAH during treatment with dasatinib [17]. Hence, we fully agree with Marc Humbert’s call for “a better understanding of the pathways involved in the efficacy and safety aspects of imatinib in order to design more targeted and better tolerated agents in the field of personalized PAH medicine” [16].

## Conclusions

In conclusion, we presume that the antiproliferative effects of long-term imatinib therapy induced by the clearly described apoptosis of pulmonary vascular smooth muscle cells [1] were the leading effect explaining the clinical course of the current patient, who could be weaned from intravenous prostanoids and attained normal pulmonary hemodynamics parameters. However, we would like to emphasize that at present such an off-label use is strongly discouraged by the authorities until more data on the risk/benefit ratio of tyrosine kinase inhibitors in patients with PAH become available.
